# Using the Unified Theory of Acceptance and Use of Technology (UTAUT) and e-health literacy(e-HL) to investigate the tobacco control intentions and behaviors of non-smoking college students in China: a cross-sectional investigation

**DOI:** 10.1186/s12889-023-15644-5

**Published:** 2023-04-25

**Authors:** Yuanyuan Ma, Mengxia Zhou, Wenli Yu, Ziyue Zou, Pu Ge, Zheng Feei Ma, Yuting Tong, Wei Li, Qiyu Li, Yunshan Li, Siya Zhu, Xinying Sun, Yibo Wu

**Affiliations:** 1 School of Public Health, Shandong University, Jinan, 250012 China; 2 School of Media and Communication, Shanghai Jiaotong University, Shanghai, 201100 China; 3 School of Foreign Languages, Weifang University of Science and Technology, Shouguang, 262700 China; 4 School of Management, Beijing University of Chinese Medicine, Beijing, 100029 China; 5 Centre for Public Health and Wellbeing, School of Health and Social Wellbeing, College of Health, Science and Society, University of the West of England, Bristol, BS16 1QY UK; 6 Department of Second Clinical Medical School, Cheeloo College of Medicine, Shandong University, Jinan, 250012 China; 7 School of Humanities and Health Management, Jinzhou Medical University, Jinzhou, 121000 China; 8 School of Public Health, Sun Yat-Sen University, Guangzhou, 510080 China; 9 School of Foreign Languages, Anhui University of Technology, Anhui, 243000 China; 10 School of Public Health, Peking University, Haidian District, 38 Xueyuan Road, Beijing, 100191 China

**Keywords:** UTAUT, E-health literacy, Tobacco control, Structural equation model, Non-smoking college students

## Abstract

**Background:**

Non-smoking college students are starting to smoke in increasing numbers, which shows that their tobacco control situation seems not optimistic. The UTAUT and e-HL are commonly used models and theories to predict health behaviors, while there are few studies on tobacco control. This paper aims to study the influencing factors of tobacco control intention and behavior of non-smoking college students in China by combining the UTAUT and e-HL.

**Methods:**

Based on the stratified sampling method, 625 college students from 12 universities were selected. Data were collected using a self-made questionnaire designed based on the UTAUT and e-health literacy scales. Data were analyzed by SPSS 22 and AMOS 26, including descriptive statistics, one-way variance analysis and structural equation model analysis.

**Results:**

The results of one-way variance analysis showed that there were significant differences in the score of non-smoking college students’ tobacco control intention or behavior by hometowns, monthly living expenses, and parents' smoking history. Performance expectancy, effort expectancy, social influence had direct positive effects on behavioral intention. Facilitating condition, behavioral intention had direct positive impacts on use behavior and e-HL had an indirect positive impact on use behavior.

**Conclusions:**

The combination of the UTAUT and e-HL can be used as an appropriate framework to predict the influencing factors of non-smoking college students' intention and behavior of tobacco control. Improving performance expectancy, effort expectancy, and e-HL among non-smoking college students, creating positive social environments, and providing facilitating condition are key aspects of increasing their tobacco control intention and behavior. It is also beneficial to promote the implementation of smoke-free campus and smoke-free family projects.

**Supplementary Information:**

The online version contains supplementary material available at 10.1186/s12889-023-15644-5.

## Introduction

Tobacco abuse has been listed by World Health Organization (WHO) as one of the top ten serious threats to human health in the twenty-first century. In terms of personal health, long-term smoking is likely to lead to cancer, emphysema, chronic bronchitis, heart disease, peptic ulcer, and other physiological diseases [1–5], and also endanger the health of others. Tobacco kills more than 8 million people every year, of which about 7 million die from diseases caused by smoking and about 1.2 million die from diseases caused by second-hand smoke exposure [6]. In 2019, the Chinese Center for Disease Control and Prevention released the National Tobacco Control Survey Report [7], pointing out that in 2018, the average initial smoking age of Chinese smokers was 20 years old. Moreover, the smoking rate in the 15–24 age group was about 17.9%, and undergraduates were at this stage. It can be seen that the number of Chinese non-smoking college students starting to smoke is gradually increasing and the situation is not optimistic. Therefore, it is very urgent to promote the intention and behavior of smoking control among non-smoking college students.

Tobacco control is globally recognized as the most effective measure for the prevention of smoking risk factors. Many countries have developed smoke-free campus policies with initial success. In the United States, for example, the number of campuses that are 100% smoke-free or have a 100% smoke-free policy has more than doubled by 2020.And numerous studies have demonstrated that smoke-free campus policies have the potential to reduce smoking among students [8–10]. At the same time, tobacco trackers were also introduced to support and promote the university smoke-free policy [11]. In addition, universities in the UK, New Zealand, and Australia have also implemented smoke-free campus policies, which have gained broad consensus among students and staff [10, 12, 13]. However, China does not have a comprehensive national smoke-free law, so Chinese schools got a late start in building smoke-free campuses [14]. Most anti-tobacco policies are newly implemented in China, which may contribute to the poor tobacco control of college students today [15]. In terms of smoke-free families, as the largest and most developed metropolis in Chinese Mainland, Shanghai's smoke-free family policy has little effect [16].

In October 2016, China put forward a new strategy called "Healthy China 2030", proposing to reduce the smoking rate to 20% by 2030 [17]. In October 2019, the Chinese government pointed out that individual tobacco control behavior referred to not smoking, not trying to smoke, not smoking in public places, quitting smoking early, and actively participating in tobacco control.

For non-smoking college students who are non-smokers in the first place, the first step of tobacco control is to continue to adhere to the non-smoking lifestyle. Moreover, in the face of various smoking incentives, such as close smoking friends, family history of smoking, and smoking roommates [18–20], they should not try smoking. At the same time, in order to reduce the dangers of second-hand smoke, non-smoking students can give full play to the role of their emotional support to people around them, and thus easier to exhort people around to reduce smoking or quit smoking [21–24]. So, the definition of tobacco control behavior of non-smoking college students in this study is to continue to insist on not smoking habits, don't try to smoke, exhort people to reduce smoking or to give up smoking, refuse to inhale second-hand smoke and actively participate in all kinds of tobacco control activities in society.

In this study, we tried to address two research questions:

- Research Question 1: What are the influencing factors of tobacco control intentions and behaviors of non-smoking college students?
- Research Question 2: Does the model have a good predictive ability for tobacco control intentions and behaviors among non-smoking college students?

## Theoretical basis and framework

In previous studies, researchers used health belief model, KAP, zero inflated models and other methods to obtain the influencing factors of tobacco control, but there were limitations such as low significance, insufficient research content and incomplete influencing factors. Therefore, it is necessary to introduce more perfect models to conduct in-depth research on tobacco control intention and behavior. Table 1 shows a specific summary of relevant studies.

**Table 1:** Previous research results and limitations

Article	Models or Methods	Main results	Limitations
Panahi R et al. (2022) [25]	Health Belief Model& Health Literacy	Perceived susceptibility, self-efficacy, and decision-making dimensions were the predictors of smoking prevention	The multiple regression analysis failed to explore the relationship between health literacy and the health belief model, which may be the reason for the few significant results
Mohmad S et al. (2022) [26]	Smoking Knowledge, Attitudes and Practices (S-KAP)	There was a significant relationship between university type and smoking attitude	No factors related to smoking behavior were found, and the influence of smoking attitude on smoking behavior was not thoroughly studied
Escario JJ et al. (2018) [27]	Zero Inflated Models	Teacher smoking on school grounds was associated with student smoking behavior	The results might not be generalized to other countries, depending on the level of tobacco exposure of students in different countries
Bast LS et al. (2021) [28]	X:IT	The X:IT II intervention did not seem to create different trajectories in current smoking among adolescents in high and low socio-economic groups	Mechanisms for preventing smoking among adolescents in high and low socio-economic groups have not been identified
Li JM et al. (2006) [29]	Multinomial Multilevel Model	Gender, age, moods and environment had positive influences on students in giving up their smoking behavior	The influencing factors were simple and have not been studied in depth

The actual usage of a technology usually depends on the user's intention to utilize it [30]. Understanding the factors that influence the intention of non-smoking college students on tobacco control will help schools take further steps to improve their behaviors and prevent them from initiating smoking. The UTAUT was proposed by Venkatesh et al. by integrating 8 theories with corresponding explanatory abilities in different fields [30]. This theory aimed to explain users' intentions and behaviors using specific information systems, and its explanatory power for users' usage behaviors was as high as 70% [31]. A review of previous studies showed that the UTAUT was a good health behavior education model, which was widely used to study physical exercise [32], reasonable sleep [33], weight loss [34] and other health behaviors. To our knowledge, there were no published studies that have applied UTAUT to the investigation of tobacco control intentions or behaviors. To gain insights into factors that may explain the influence of tobacco control, this study analyzed the tobacco control intentions of non-smoking college students and their tobacco control behaviors, as well as the relationship between these factors. This model integrated the following factors [30]:

- 1. Performance Expectancy (PE) refers to “the degree to which an individual believes that using the system will help him or her to attain gains in job performance”.
- 2. Social Influence (SI) refers to “the degree to which an individual perceives those important others believe he or she should use the new system”.
- 3. Effort Expectancy (EE) refers to “the degree of ease associated with the use of the system”.
- 4. Facilitating Conditions (FC) refers to “the degree to which an individual believes that an organizational and technical infrastructure exists to support the use of the system”.

**Fig. 1:**
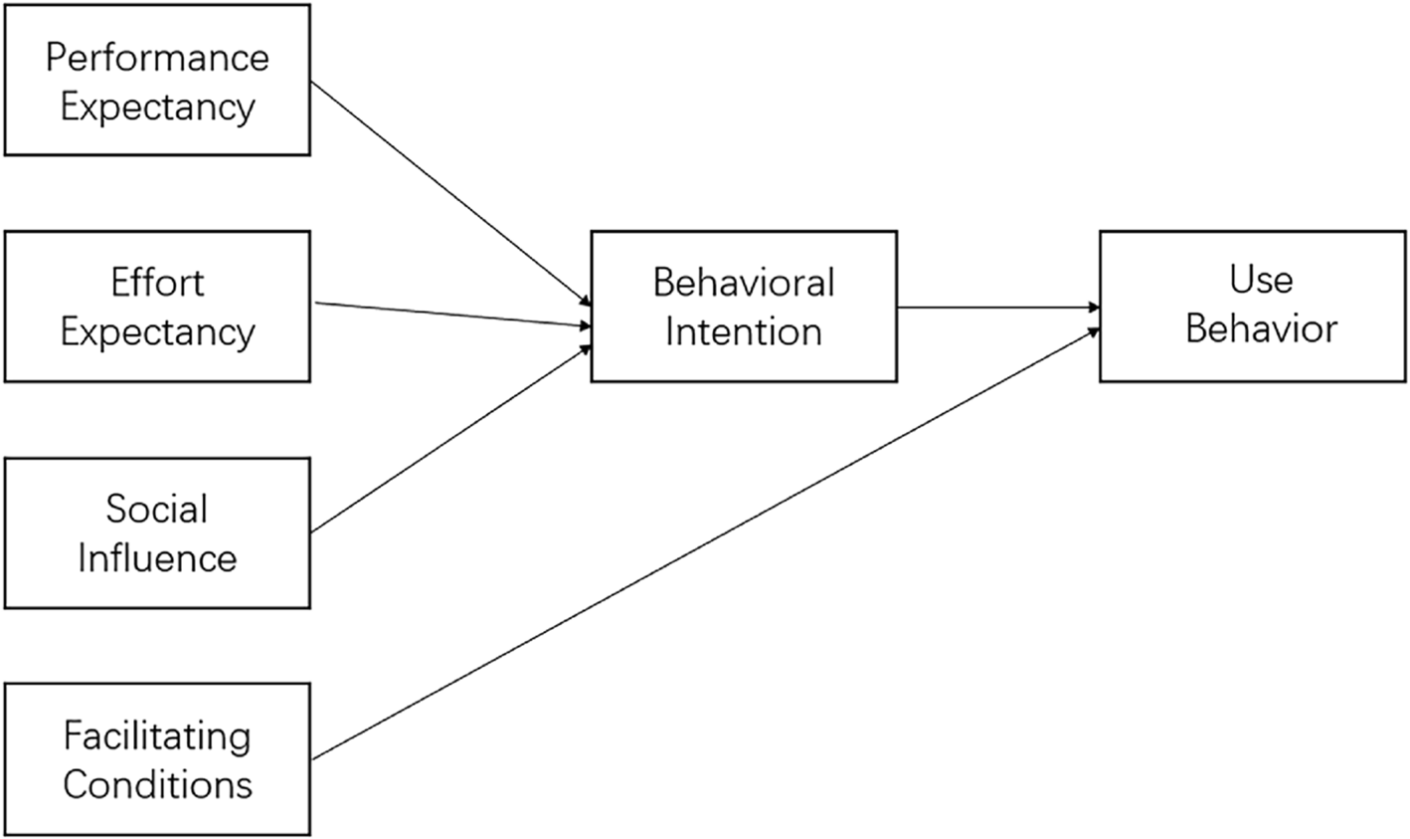
UTAUT Model

In the UTAUT (Fig. 1), performance expectancy, effort expectancy, and social influences are directly associated with behavioral intentions while the final facilitating conditions are associated with actual usage.

Liu, Yong-Bing et al. have found that health literacy (HL) level is significantly correlated with smoking status [35]. Low HL is closely associated with smoking [36–38], smoking relapse [39] and weak smoking cessation program outcomes [40], while increasing HL levels can change people's smoking-related behaviors [41]. At the same time, studies have shown that more than one-third of the students have insufficient HL [25, 42]. Other studies have confirmed that smoking control intervention measures based on network could improve the tobacco control rate [43, 44]. On the one hand, the cost of obtaining tobacco control information through the Internet was lower than that of clinical intervention, and it was more attractive to users [45]. On the other hand, college students as the main force of Internet use could access the Internet anytime and anywhere to inquire about smoking treatment information [46]. Therefore, on the basis of HL and considering the role of online knowledge, this study adopted e-health literacy (e-HL) to measure. E-HL not only required people to fully obtain the information that they need, while also required them to think critically about the quality of electronic health information, namely the ability to distinguish good from bad [47]. At present, e-HL is becoming an effective measurement and intervention tool to promote the health behaviors of ordinary people [48]. In order to promote non-smoking college students to develop good tobacco control behaviors, it is necessary to study the influence of e-HL on tobacco control of non-smoking college students and methods to improve the e-HL of Internet users.

In the UTAUT model, the facilitating condition refers to "the degree to which individuals believe that organizational and technical infrastructure exists to support the use of the system" [30], that is, the degree to which non-smoking students believe that the currently available tobacco control information and other conditions exists to support the adoption of tobacco control behaviors. Since e-HL emphasizes the acquisition of online information, it can be regarded as the basis of facilitating conditions. Only if non-smoking college students are able to access online health information can they use it to support their tobacco control behavior. In other words, e-HL influences tobacco control behavior through facilitating condition. It can be seen that combining UTAUT with e-HL seems to help better understand the factors influencing college students' intention and behavior to control smoking, thus further enhancing the role of the model in promoting smoking prevention. At the same time, the combination of UTAUT and e-HL can overcome the limitations of previous studies in which the relationship between the role of each factor of the model is unclear and the factors influencing behavioral intention and actual behavior are not studied in depth (Table 1).

**Fig. 2:**
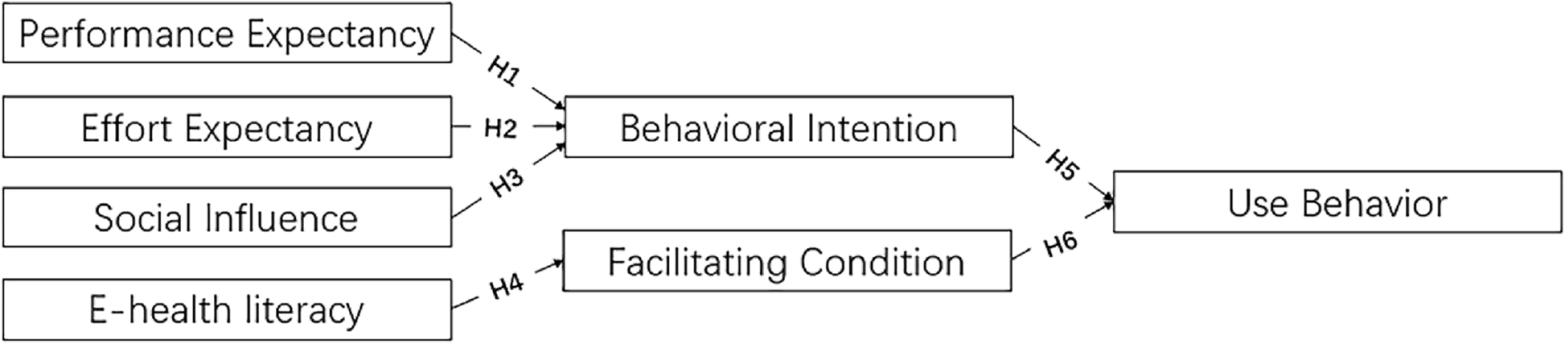
Theoretical model and hypothesis

The model diagram (Fig. 2) is as follows:

## Methods

### Study design and sample

This study was a cross-sectional study conducted among Chinese college students from June to December 2021. Considering the obvious stratification of educational resources and educational levels among universities in China, the use of stratified sampling can make the samples more representative and reduce sampling errors. In this study, 12 universities of different levels nationwide were selected through a stratified random sampling method, including 4 "double first-class" universities, 4-first batch of universities and 4-s batch universities. The proposed stratified random sampling process was shown in Table 2. Investigators were recruited among these 12 universities for training and distributing questionnaires. In order to ensure the representativeness of the samples, we required every investigator to use the quota sampling method to recruit respondents, which means that the proportion of gender and household registration of college students involved in the investigation is roughly 1:1. The inclusion criteria include: (1) being an undergraduate student;(2) living and studying in China for more than 5 years; (3) being willing to participate in the study and sign the informed consent form. The exclusion criteria include:(1) smoking college students; (2) having serious heart, liver, kidney and other organ diseases or mental disorders;(3) having severe aphasia, apraxia and cognitive dysfunction;(4) having taken part in similar studies recently.

**Table 2:** Proposed stratified sampling based on educational level

Strata	Educational Level	Eligible Universities	Selected Universities	Names of Universities	Surveys per Universities	Surveys per stratum
Stratum 1	"Double first-class" universities ^a^	147	4	Peking University Shandong University Jilin University Tianjin University	20–30	80–120
Stratum 2	first batch of universities ^b^	458^d^	4	Shanxi Medical University Yantai University University of Jinan Shanxi University	60–80	240–320
Stratum 3	second batch universities ^c^	431	4	Guangxi University of Chinese Medicine Ludong University Yunnan University of Chinese Medicine Xi’an International University	60–80	240–320

^a^ “Double first-class” universities refer to world-class universities and first-class discipline construction universities

^b^First batch of universities refer to the first batch of undergraduates admitted to the national unified examination for admission to ordinary colleges and universities

^c^Second batch universities refer to the second batch of undergraduates admitted to the national unified examination for admission to ordinary colleges and universities

^d^Not including the “double first-class” universities

### Data collection

**Fig. 3:**
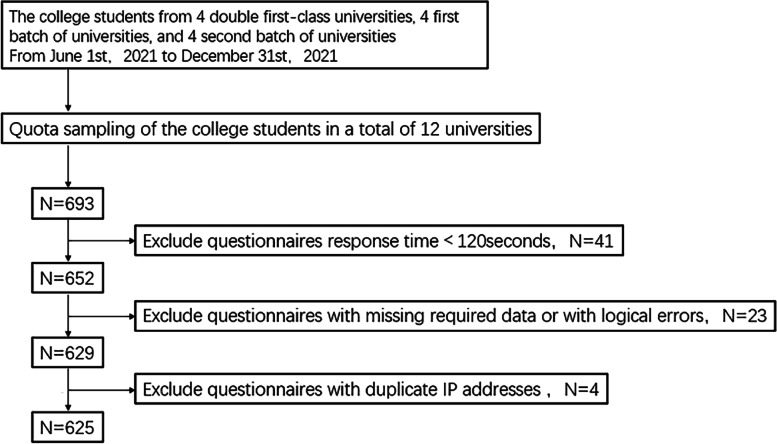
Participant inclusion process

We recruited 1–3 investigators in each university. And each investigator was required to distribute and recover 20–30 online questionnaires on a one-to-one basis. Before the investigation, investigators received standardized training about the content and precautions of the questionnaire to ensure the rigor of the questionnaire distribution process. The exclusion criteria were shown in Fig. 3. Finally, 625 valid questionnaires were obtained with an effective rate of 90.2%.

### Measures and variables

At the early stage of the study, the questionnaire was initially designed through literature search and group discussion. The main measuring part of this study consisted of the UTAUT scale and the e-HL scale. The UTAUT scale was developed by referring to the subfactors of the UTAUT scale designed by Venkatesh et al. in 2003 [30] and incorporating the actual situation of tobacco control among non-smoking college students in China. The UTAUT factors were measured with a total of 6 dimensions and 19 items (Appendix). For the e-HL scale we used the mobile-eHealth Literacy Scale developed by Chinese scholars Ying-min W et al. with 3 dimensions and 12 items [49] (Appendix). All 31 questions were measured on a five-point Likert scale from 1 (very disagree) to 5 (very agree).

A purposive sampling method was used for forming the panel of experts. Expert was included in the following criteria: had a postgraduate degree in health administration, health policy, health informatics, information system; had published work on the subject; at least 5 years professional knowledge and experience in the field of tobacco control. Individuals who had not published a paper on the research topic for more than five years were excluded. Finally, 11 experts were invited to participate in the evaluation of this questionnaire through email. Subsequently, a preliminary survey was conducted among undergraduates. According to the experts’ opinions and the results of 50 pre-surveys, the questionnaire has been revised and improved repeatedly to ensure the rationality and scientificity of the questionnaire.

The reliability and validity results of each dimension were shown in Table 3. It could be seen that all the measurement indexes had reached the standard range.

**Table 3:** Reliability and validity of the questionnaire

Factors	Variables	I-CVI	Pc	k	Rating ^a^
PE	PE1	1.000	0.0005	1.00	Excellent
	PE2	0.818	0.0269	0.81	Excellent
	PE3	1.000	0.0005	1.00	Excellent
	^b^ Cronbach’s Alpha = 0.873, ^c^ CR = 0.884, ^d^ AVE = 0.719				
EE	EE1	0.909	0.0054	0.91	Excellent
	EE2	0.909	0.0054	0.91	Excellent
	EE3	1.000	0.0005	1.00	Excellent
	Cronbach’s Alpha = 0.847, CR = 0.858, AVE = 0.668				
SI	SI1	1.000	0.0005	1.00	Excellent
	SI2	1.000	0.0005	1.00	Excellent
	SI3	1.000	0.0005	1.00	Excellent
	SI4	0.818	0.0269	0.81	Excellent
	Cronbach’s Alpha = 0.852, CR = 0.821, AVE = 0.536				
FC	FC1	1.000	0.0005	1.00	Excellent
	FC2	0.909	0.0054	0.91	Excellent
	FC3	0.818	0.0269	0.81	Excellent
	Cronbach’s Alpha = 0.832, CR = 0.723, AVE = 0.561				
BI	BI1	1.000	0.0005	1.00	Excellent
	BI2	1.000	0.0005	1.00	Excellent
	BI3	1.000	0.0005	1.00	Excellent
	Cronbach’s Alpha = 0.862, CR = 0.856, AVE = 0.666				
UB	UB1	1.000	0.0005	1.00	Excellent
	UB2	1.000	0.0005	1.00	Excellent
	UB3	1.000	0.0005	1.00	Excellent
	Cronbach’s Alpha = 0.892, CR = 0.891, AVE = 0.731				
e-HL	SP1	1.000	0.0005	1.00	Excellent
	SP2	1.000	0.0005	1.00	Excellent
	SP3	1.000	0.0005	1.00	Excellent
	IA1	1.000	0.0005	1.00	Excellent
	IA2	1.000	0.0005	1.00	Excellent
	IA3	0.909	0.0054	0.91	Excellent
	IA4	0.909	0.0054	0.91	Excellent
	IA5	0.909	0.0054	0.91	Excellent
	IJ1	0.818	0.0269	0.81	Excellent
	IJ2	1.000	0.0005	1.00	Excellent
	IJ3	1.000	0.0005	1.00	Excellent
	IJ4	1.000	0.0005	1.00	Excellent
	Cronbach’s Alpha = 0.906, CR = 0.912, AVE = 0.776				

*I-CVI* Item Content Validity Index > 0.78, *Pc* probability of random agreement, *k* modified kappa coefficient obtained by designing the relevant proportion of agreements

^a^Evaluation criteria of k: poor ≤ 0.39, weak = 0.40–0.59; good = 0.60–0.73; excellent ≥ 0.74 according to Fleiss [50]; S-CVI: I-CVI average of the items in the subscale

^b^Cronbach’s Alpha > 0.80

^c^ *CR* Composite Reliability > 0.70

^d^ *AVE* Average Variance Extracted > 0.50

### Statistical methods

In this study, SPSS 26 was used for exploratory analysis, and AMOS 24 was used for structural equation model analysis. Statistical significance was set at *P* < 0.05. First, we obtained frequency (N) and percentage (%) statistics to show the characteristics of non-smoking college students. Second, we obtained mean (M) and standard deviation (SD) statistics to show the scores of the UTAUT and e-HL in each dimension, and conducted a one-way variance analysis to examine the differences in scores of each dimension among non-smoking college students with different characteristics. Lastly, the structural equation model (SEM) was used to verify the influence path of each factor on non-tobacco college students' behavioral intention and use behavior of tobacco control. Maximum likelihood estimation was performed to estimate these parameters in SEM. (Note: Scores of the UTAUT and e-HL in all dimensions were conformed to normal distribution).

## Results

### Basic characteristics of participants

Among the 625 participants, 41.1% were males, 52.0% were registered urban residents, and 94.6% had no religious belief. The majority of their major, ethnicity, and monthly living expenses were engineering (29.9%), Han (92.3%), and 145.1–217.6 dollars per month (44.3%) respectively. The smoking percentage of their fathers was 45.9%, while that of mothers was 1.9%. (Table 4).

**Table 4:** Basic characteristics of non-smoking college students

Characteristics	Group	Number(N)	Percentage (%)
Gender	Male	257	41.1
	Female	368	58.9
Major	Engineering	187	29.9
	Medicine	162	25.9
	Sciences	69	11.0
	Economics	39	6.2
	Arts	56	9.0
	Management	47	7.5
	Others	65	10.4
Last year’s grade ranking	Top 10%	113	18.1
	11%-20%	124	19.8
	21%-30%	81	13.0
	31%-40%	86	13.8
	41%-50%	82	13.1
	51%-60%	79	12.6
	> 60%	60	9.6
Household registration	Urban areas	325	52.0
	Rural areas	300	48.0
Ethnicity	Han	577	92.3
	Non-Han	48	7.7
Religion	Yes	34	5.4
	No	591	94.6
Monthly living expenses (yuan)^*^	≤ 145.1(≤ 1000)	136	21.8
	145.1–217.6(1000–1499)	277	44.3
	217.7–290.1 (1500–1999)	124	19.8
	≥ 290.3(≥ 2000)	88	14.1
Father's smoking experience	Never smoke	244	39.0
	Smoke	287	45.9
	Quitted	94	15.0
Mother's smoking experience	Never smoke	609	97.4
	Smoke	12	1.9
	Quitted	4	0.6

1 USD = 6.89CNY

### One-way variance analysis of college students with different characteristics in each dimension

The mean scores of performance expectancy, effort expectancy, social influence, facilitating conditions, behavioral intention, use behavior and e-HL for the participants were respectively 12.03(total15),11.83(total15), 14.87(total20), 11.60(total15), 11.29(total15), 11.58(total15), 45.98(total60). There were significant differences in the scores of performance expectancy, facilitating condition, use behavior, and e-HL by hometown (*P* < 0.05), and the non-smoking college students’ scores with rural registered residence were significantly lower than those with urban registered residence. There were significant differences in the scores of facilitating condition, behavioral intention, use behavior, and e-HL by monthly living expenses (*P* < 0.05). And the scores of college students with higher monthly living expenses were also higher than those with monthly living expenses < 144.7 dollars. It meant that the hometown and the level of living expenses had an impact on the tobacco control of non-smoking college students. That’s to say, non-smoking college students with high living standards had better tobacco control behaviors. There were also significant differences in the scores of social influences, facilitating condition, use behavior, and e-HL by fathers’ smoking experience (*P* < 0.05). And the average score from the highest to the lowest were students whose fathers had never smoked, had quit smoking, and smoke. However, there were significant differences in the scores of performance expectancy, effort expectancy, facilitating condition, and use behavior by mother’s smoking experience. It showed that the smoking behavior of parents in the family had a significant impact on their child's tobacco control behavior (Table 5).

**Table 5:** One-way variance analysis of different characteristics (M, SD)

	PE	EE	SI	FC	BI	UB	e-HL
Total	12.03(2.28)	11.83(2.57)	14.87(3.03)	11.60(2.23)	11.29(2.38)	11.58(2.25)	45.98(8.34)
Household registration							
Urban areas	12.25(2.32)	11.98(2.53)	15.09(3.13)	11.87(2.30)	11.41(2.51)	11.85(2.27)	46.82(8.76)
Rural areas	11.80(2.23)	11.67(2.61)	14.64(2.91)	11.30(2.11)	11.17(2.22)	11.30(2.20)	45.06(7.76)
* F* value	6.011	2.242	3.342	10.180	1.497	9.541	7.032
* P* value	0.014	0.135	0.068	0.001	0.222	0.002	0.008
Monthly living expenses (CNY)							
≤ 1000	11.90(2.29)	11.36(2.76)	14.45(2.97)	10.93(2.37)	10.84(2.55)	11.01(2.53)	43.77(8.19)
1000–1499	12.12(2.17)	11.93(2.47)	14.86(2.95)	11.63(2.09)	11.35(2.17)	11.53(2.02)	46.15(7.93)
1500–1999	12.00(2.56)	12.10(2.63)	15.11(3.19)	11.91(2.22)	11.74(2.41)	12.06(2.25)	47.76(8.42)
≥ 2000	11.99(2.24)	11.90(2.48)	15.24(3.14)	12.08(2.21)	11.20(2.59)	11.97(2.28)	46.34(9.08)
* F* value	0.305	2.119	1.581	6.410	3.254	5.898	5.261
* P* value	0.821	0.097	0.193	0.000	0.021	0.001	0.001
Mothers’ smoking experience							
Never smoke	12.04(2.27)	11.88(2.54)	14.91(3.04)	11.63(2.19)	11.32(2.36)	11.62(2.22)	46.10(8.35)
Smoke	10.67(2.71)	10.58(3.20)	14.08(2.81)	10.33(3.39)	10.42(3.20)	10.50(3.26)	42.33(6.16)
Quitted	14.25(0.96)	8.25(2.50)	12.00(1.41)	10.00(2.16)	9.50(1.73)	9.50(1.73)	39.00(8.76)
* F* value	4.086	5.478	2.254	3.061	2.009	3.206	2.624
* P* value	0.017	0.004	0.106	0.048	0.135	0.041	0.073
Fathers’ smoking experience							
Never smoke	12.17(2.17)	12.04(2.46)	15.31(2.98)	11.83(2.09)	11.50(2.43)	11.89(2.21)	46.93(8.17)
Smoke	11.86(2.44)	11.69(2.66)	14.59(3.14)	11.36(2.41)	11.15(2.44)	11.33(2.38)	45.09(8.80)
Quitted	12.21(2.03)	11.73(2.56)	14.60(2.69)	11.71(1.90)	11.20(2.00)	11.57(1.83)	46.21(7.01)
* F* value	1.574	1.311	4.141	3.152	1.454	4.155	3.267
* P* value	0.208	0.270	0.016	0.043	0.234	0.016	0.039

### Path relationships among all dimensions

**Fig. 4:**
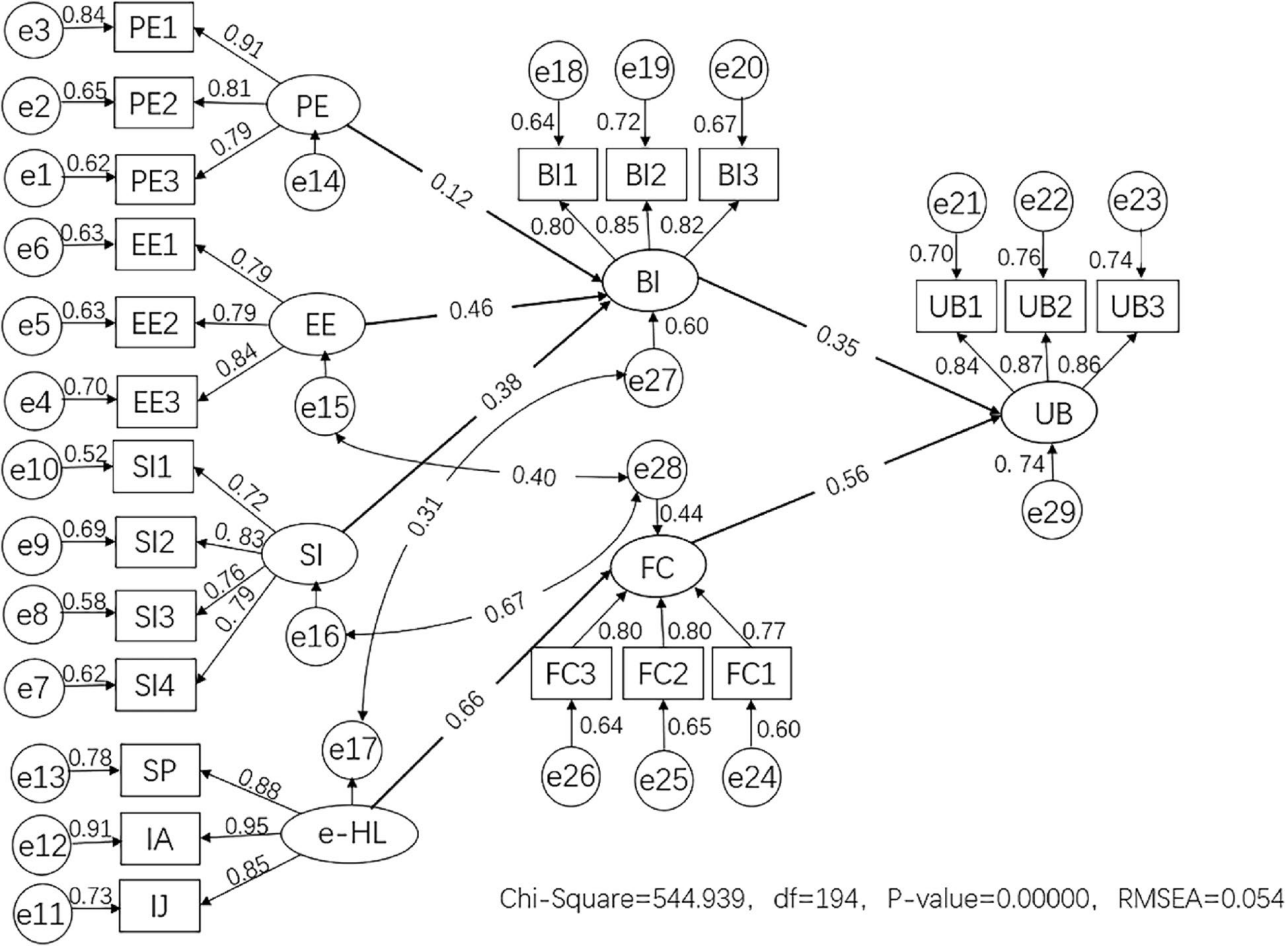
SEM of influencing factors of tobacco control intention and behavior among non-smoking college students

The final model was obtained by fitting and modifying the initial model. (Fig. 4) The model fit indices of the SEM were all within specifications (*χ* ^2^/df = 2.809 < 3, RMSEA = 0.054 < 0.08, GFI = 0.927, AGFI = 0.905, NFI = 0.944, CFI = 0.963, TLI = 0.956, IFI = 0.963, RFI = 0.933), indicating good model fit. The corresponding standardized path coefficients and significance were as follows. (Table 6) Performance expectancy (*r* = 0.117, *P* < 0.01), effort expectancy (*r* = 0.462, *P* < 0.001), social influence (*r* = 0.380, *P* < 0.001) had direct positive effects on behavioral intention. Facilitating condition (*r* = 0.561, *P* < 0.001) and behavioral intention (*r* = 0.354, *P* < 0.001) had direct positive effects on use behavior. The indirect effect of one dimension on another dimension was equal to the product of the regression coefficients of two directly related dimensions, so e-HL (*r* = 0.373, *P* < 0.001) had an indirect positive effect on use behavior.

**Table 6:** Standardized path coefficients for the final model

	Relationship			Estimate	*P*	SE	CR	Result
H1	PE	→	BI	0.117	.001	0.039	3.275	Supported
H2	EE	→	BI	0.462	< .001	0.043	9.656	Supported
H3	SI	→	BI	0.380	< .001	0.047	8.129	Supported
H4	e-HL	→	FC	0.664	< .001	0.011	15.423	Supported
H5	BI	→	UB	0.354	< .001	0.051	6.442	Supported
H6	FC	→	UB	0.561	< .001	0.058	9.848	Supported

## Discussion

Through the UTAUT model and e-HL, the influencing factors of tobacco control intention and behaviors of non-smoking college students were studied. It was found that the actual score/total score of six dimensions in the UTAUT model and e-HL ranged from 0.734 to 0.802. There were significant differences in smoking control intentions or behaviors among non-smoking college students with different hometowns and monthly living expenses. And whether parents smoked also affected children's tobacco control intentions or behaviors. The structural equation model found that performance expectancy, effort expectancy, and social influence directly affected tobacco control intention, and then indirectly affected tobacco control behavior, facilitating conditions directly affected tobacco control behavior of college students, while e-HL indirectly affected tobacco control behavior through facilitating conditions.

First of all, we found that the average score/total score of social influence in the six dimensions of the UTAUT model was the lowest. On the one hand, it indicated that people around college students paid little attention to tobacco control. Studies have proved that friends, classmates, and parents can influence the onset of smoking in college students [51]. In particular, some non-smoking college students started smoking because of a smoking close friend, a smoking family member [52, 53]. On the other hand, the publicity and education of tobacco control in schools were not in place, so non-smoking college students did not realize the importance of tobacco control. Although many schools had smoke-free campus policies, these policies were not implemented. Many students, staff and faculty still smoked [54]. Coupled with the poor implementation of smoke-free policies on campus by university staff, non-smoking college students were more likely to deliberately ignore them [13, 55, 56] or even maintain a negative attitude towards these policies [57]. So, it’s necessary to promote the publicity and education of the smoke-free policy on campus, strengthen the implementation of the smoke-free policy, and form a good habit of tobacco control among non-smoking college students, so as to form a positive social influence.

The results of the one-way variance analysis of college students with different characteristics in various dimensions showed that hometowns and monthly living expenses had impacts on tobacco control behaviors of non-smoking college students. And non-smoking college students with lower living standards also had a lower level of tobacco control behaviors, consistent with the findings of a U.S. study [58]. This might be the result of low economic conditions contributing to students' feelings of inferiority and isolation [59–61], which in turn led to ineffective social skills, poor sleep quality, and reduced emotional and health status [62]. These negative conditions were more likely to cause college students to start trying harmful habits such as smoking and drinking [63, 64]. Furthermore, smoking behavior of people with lower socioeconomic status was generally more common and persistent, and this situation had greatly led to health inequality based on socioeconomic status [65–70]. It involved the allocation of social medical and health resources [71], legal and policy reforms, global health cooperation and other aspects. It was also found that parental smoking was more likely to be a trigger for non-smoking students to have their first cigarette. Another study had the same results [72]. In addition, a study in Thailand showed that undergraduate students whose parents considered that cigarette smoking was acceptable and those who were uncertain about their parents' concerns on cigarette smoking had a higher likelihood of cigarette smoking than those whose parents considered that cigarette smoking was unacceptable [73]. Hence, social environmental factors, such as family members' smoking attitudes and behaviors, were important determinants of whether non-smoking college students try smoking, and college students may perceive smoking to be acceptable from family members' smoking behaviors. This suggested the value of maintaining strict "no-smoking" norms at home and fostering better nonsmoking attitudes [74].

Each path in the structural equation model was significant. Effort expectancy and social influence had greater impacts on behavioral intention. Effort expectancy here referred to the ease of obtaining, understanding, and using tobacco control information online. Effort expectancy was closely related to the intensity of tobacco control intention. As some researches showed that website ease of use was one of the main determinants of personal satisfaction with the website and willingness to participate [75, 76]. Dotson JAW et al. also found that when developing an iPad app to help pregnant women control tobacco, the easier the information was to understand, the more it helped them understand the risks of tobacco use [77]. All these reflected that the ease of use of tobacco control information could improve people's willingness to control tobacco. This prompted the necessity to focus on the importance of training college students to search and utilize tobacco control information online. At the same time, authors of online tobacco control articles should try to make their essays less difficult. Giving examples, making analogies, and applying props appropriately are all techniques to keep the articles from being raw. The social influence had a significant impact on behavioral intention, which was the same as the one-way variance analysis. Facilitating conditions had the greatest influence on use behaviors, indicating providing personalized tobacco control information and relevant help could directly promote behaviors. And e-HL in the structural equation by facilitating conditions had an indirect effect on use behavior. It meant that college students with adequate e-health literacy could get high-quality and personalized tobacco control information and thus became a facilitating condition for tobacco control. According to a 2018 Saudi Arabian study [78], literacy in media use was associated with tobacco and alcohol use. It was crucial to provide college students with adequate resources and counsel on their plight by establishing a dedicated knowledge base and counseling channels both online and offline. As a result, in order to achieve tobacco control of non-smoking college students, it is necessary to improve their effort expectancy, performance expectancy, social influence and facilitating conditions and e-HL. The information platform should provide accurate, scientific and easy-to-understand knowledge of tobacco control, so as to give full play to the role of network information. Schools and families should create a good environment to correctly guide college students' concepts and attitudes toward tobacco control. And students themselves could improve their own e-health literacy [79] and identify suitable ways to control tobacco.

The advantage of this study lay in three aspects: the combination of the UTAUT and e-HL for the first time to study the intention and behaviors of tobacco control of non-smoking college students, the innovative application of the UTAUT model to the information-related health behavior of tobacco control, and the full consideration of the influence of e-HL on non-smoking college students' tobacco control through network information.

## Limitation and future research

However, the research still had several limitations. First, the relationship between e-HL and other dimensions of the UTAUT was not studied which needed to search more information. Second, most of the samples were from students with engineering and medicine majors, and students in the top 50% of their grades accounted for more, which may lead to higher scores than the actual scores. Third, the data were obtained through the self-report questionnaire, which may lead to information recall bias. Fourth, this study was a cross-sectional study, and the causal relationship between various dimensions could not be determined. Nevertheless, this study still provided some valuable results and conclusions for promoting college students' intention and behavior of tobacco control through the combination of the UTAUT and e-HL.

## Conclusion

We obtained an appropriate framework to predict and intervene in non-smoking college students' tobacco control intention and behavior to promote their continued adherence to non-smoking and non-attempt smoking. Improving performance expectancy, effort expectancy, and e-HL among non-smoking college students, creating positive social environments, and providing facilitating condition are key aspects of increasing their tobacco control intention and behavior. This provides a basis for society, schools and families to help non-smoking college students to control smoking, and helps to take targeted and efficient measures to promote the implementation of smoke-free families and schools.

## Inferences

Based on the results of this study, we make the following recommendations: The education programs require the joint efforts of schools, families and society. The school should continue to promote the "smoke-free campus" policy, strengthen the implementation, and develop special education plans to improve the e-HL and tobacco control ability of college students; In terms of families, campus should publicize smoke-free family plans to non-smoking college students' parents to reduce incentives for college students to smoke their first cigarette. Society should provide a good tobacco control environment and easy-to-understand, simple and practical tobacco control information, so as to promote non-smoking college students do not smoke and do not try to smoke.

## Supplementary Information

**Additional file 1.**

## Data Availability

The data used and analyzed during the study were available from the corresponding author on reasonable request.
