# Supplementary material for: Using the Unified Theory of Acceptance and Use of Technology (UTAUT) and e-health literacy(e-HL) to investigate the tobacco control intentions and behaviors of non-smoking college students in China: a cross-sectional investigation
Source: BMC Public Health. 2023 Apr 25;23:765. doi: 10.1186/s12889-023-15644-5 (PMC10127360; doi:10.1186/s12889-023-15644-5)
Supplement: Supplementary file 1 — Additional file 1. [file 12889_2023_15644_MOESM1_ESM.docx]

**The UTAUT scale measurement items**

| Factors | Variables | Items | Subfactors |
| --- | --- | --- | --- |
| Performance Expectancy  (PE) | PE1 | I believe that using this information effectively can improve the efficiency of my tobacco control. | Perceived usefulness |
|  | PE2 | Effectively using this information for tobacco control can increase my ability of tobacco control and information utilization. | Extrinsic Motivation |
|  | PE3 | Using this information effectively for tobacco control is more effective than other methods. | Relative advantage |
| Effort Expectancy  (EE) | EE1 | I find it easy to get this information. | Ease of use |
|  | EE2 | Understanding this information is easy for me. | complexity |
|  | EE3 | It would be easy for me to use this kind of information for tobacco control. | perceived ease of use |
| Social Influence  (SI) | SI1 | The people around me attach great importance to the analysis and utilization of information related to tobacco control. | Subjective norm |
|  | SI2 | The people around me are very supportive of the analysis and utilization of information related to tobacco control. | Social factors |
|  | SI3 | The environment I live in encourages me to get information about tobacco control. |  |
|  | SI4 | Being able to analyze and use this information is seen as a manifestation of personal ability. | Image |
| Facilitating Condition  (FC) | FC1 | It is easy for me to make use of tobacco control information if there are resources available. | Perceived behavioral control |
|  | FC2 | Using this information fits my tobacco control style. | Compatibility |
|  | FC3 | I can get help in solving difficulties about information collecting and utilization. | Facilitating conditions |
| Behavioral Intention  (BI) | BI1 | I intend to continue to use this information for tobacco control. |  |
|  | BI2 | I intend to continue to use this information for tobacco control. |  |
|  | BI3 | I intend to recommend this approach to tobacco control to other people. |  |
| Use Behavior  (UB) | UB1 | I can collect and filter this information to control tobacco. |  |
|  | UB2 | I can use this kind of information to help me complete my tobacco control. |  |
|  | UB3 | I can recommend this approach to tobacco control that emphasizes information collection and utilization to others. |  |

**The e-HL scale measurement items**

| Factors | Variables | Items |
| --- | --- | --- |
| Self-Perception  (SP) | SP1 | The Internet helps me a lot in getting healthy resources. |
|  | SP2 | I know how to use the Internet to answer my health questions. |
|  | SP3 | I am confident in using information from Internet to make health decisions. |
| Information Acquisition  (IA) | IA1 | I know how to find helpful health resources on the Internet. |
|  | IA2 | I know where to find helpful health resources on the Internet. |
|  | IA3 | In addition to computers, I also use network mobile devices (such as mobile phones) to obtain health information. |
|  | IA4 | Even if it's credible, high-quality health information, I also carefully consider whether it is suitable for my particular situation |
|  | IA5 | I will use the Internet to actively learn about health-related knowledge. |
| Interactive Judging  (IJ) | IJ1 | I will follow or participate in a health-related forum, QQ group, or WeChat group. |
|  | IJ2 | I can distinguish high quality from low quality health resources on the Internet. |
|  | IJ3 | I have skills to evaluate whether the health resources on the Internet are good or not. |
|  | IJ4 | I can clearly and completely provide the diagnosis information to the doctor on the Internet (such as disease description, past |
|  |  | medical records, physiological data, etc.) |
